# Setting health research priorities using the CHNRI method: IV. Key conceptual advances

**DOI:** 10.7189/jogh-06-010501

**Published:** 2016-06

**Authors:** Igor Rudan

**Affiliations:** 1 Centre for Global Health Research, The Usher Institute for Population Health Sciences and Informatics, The University of Edinburgh, Edinburgh, Scotland, UK

## Abstract

**Introduction:**

Child Health and Nutrition Research Initiative (CHNRI) started as an initiative of the Global Forum for Health Research in Geneva, Switzerland. Its aim was to develop a method that could assist priority setting in health research investments. The first version of the CHNRI method was published in 2007–2008. The aim of this paper was to summarize the history of the development of the CHNRI method and its key conceptual advances.

**Methods:**

The guiding principle of the CHNRI method is to expose the potential of many competing health research ideas to reduce disease burden and inequities that exist in the population in a feasible and cost–effective way.

**Results:**

The CHNRI method introduced three key conceptual advances that led to its increased popularity in comparison to other priority–setting methods and processes. First, it proposed a systematic approach to listing a large number of possible research ideas, using the “4D” framework (description, delivery, development and discovery research) and a well–defined “depth” of proposed research ideas (research instruments, avenues, options and questions). Second, it proposed a systematic approach for discriminating between many proposed research ideas based on a well–defined context and criteria. The five “standard” components of the context are the population of interest, the disease burden of interest, geographic limits, time scale and the preferred style of investing with respect to risk. The five “standard” criteria proposed for prioritization between research ideas are answerability, effectiveness, deliverability, maximum potential for disease burden reduction and the effect on equity. However, both the context and the criteria can be flexibly changed to meet the specific needs of each priority–setting exercise. Third, it facilitated consensus development through measuring collective optimism on each component of each research idea among a larger group of experts using a simple scoring system. This enabled the use of the knowledge of many experts in the field, “visualising” their collective opinion and presenting the list of many research ideas with their ranks, based on an intuitive score that ranges between 0 and 100.

**Conclusions:**

Two recent reviews showed that the CHNRI method, an approach essentially based on “crowdsourcing”, has become the dominant approach to setting health research priorities in the global biomedical literature over the past decade. With more than 50 published examples of implementation to date, it is now widely used in many international organisations for collective decision–making on health research priorities. The applications have been helpful in promoting better balance between investments in fundamental research, translation research and implementation research.

Child Health and Nutrition Research Initiative (CHNRI) started as an initiative of the Global Forum for Health Research in Geneva, Switzerland [1]. Its aim was to develop a method that could assist priority setting in health research investments [2]. The first version of the CHNRI method was published in 2007–2008 [3–6]. The aim of this paper was to summarize the history of the development of the CHNRI method and its key conceptual advances [7].

## The history of the development of the CHNRI method

In 2005, CHNRI was funded by the World Bank to develop a method that could assist priority setting in health research investments. In March 2005, Professor Robert E. Black, from Johns Hopkins University in Baltimore, USA, Dr Shams El Arifeen, Director of the CHNRI Secretariat from the International Centre for Diarrheal Disease Research (ICCDR,B) in Dhaka, Bangladesh, and Nancy Hughart, Secretary of the CHNRI office met in Geneva and appointed me to lead the process of methodology development for CHNRI. Professors Jennifer Bryce and Robert E. Black from the Child Health Epidemiology Reference Group (CHERG) recommended me for this role based on my previous work and contributions to CHERG. In May 2005, at a meeting at Johns Hopkins University, I presented the first background review on different approaches to research priority setting and an early conceptual framework for the future CHNRI method. I received feedback from world–renowned experts in global health, such as Professors Dean Jamison, Ok Pannenborg and Mary Ann Lansang; and from priority–setting experts Jennifer Gibson, Lydia Kapiriri and Craig Mitton.

In June 2005, assisted by the new CHNRI secretary, Ms Deborah Horner, I invited a larger group of global health experts to Dubrovnik, Croatia, to help me develop the method further and plan its implementation in several fields of global health: newborn health (Joy E. Lawn and Zulfiqar A. Bhutta), childhood pneumonia and diarrhea (Harry Campbell and Claudio F. Lanata), child development (Maureen Black and Julie Meeks Gardner), childhood accidents (Shanthi Ameratunga and Adnan A. Hyder) and zinc (Kenneth H. Brown and Sonja Y. Hess). In September 2005, at the *9^th^ Annual Meeting of the Global Forum for Health Research* in Mumbai, India, I presented the first draft version of the CHNRI method and an example of its application in the field of childhood pneumonia. I did this together with Dr Shams El Arifeen, Professor Robert E. Black and Professor Harry Campbell, who were mentoring and supporting me throughout the process of methods development. In December 2005, at the launch of the *Child Survival: Countdown to 2015* conference in London, UK, I presented the key concepts of the new CHNRI method at the plenary session on health research agenda for child survival. Following the feedback from the audience, I revised and improved the method.

In April 2006, I visited Cape Town to conduct the first national–level implementation of the CHNRI exercise – to set research priorities for child health in South Africa. I was supported by Dr Mickey Chopra and Dr Mark R. Tomlinson, from MRC’s Health Systems Unit in Cape Town. At this point, the first exercises on childhood pneumonia and zinc were already being piloted at the global level, by Professor Harry Campbell from the University of Edinburgh, UK and Professor Kenneth Brown from the University of California in Davis, USA. At this point, I suggested that two more consultants should be contracted to assist me with preparations for publishing a series of four papers that would describe the CHNRI method: Drs Jennifer L. Gibson and Lydia Kapiriri from the University of Toronto.

In May 2006, the CHNRI Foundation organized a meeting at Johns Hopkins University. The meeting had a wider participation, aiming to include several representatives from donor agencies who could potentially be interested in the implementation of the method. The most recent version of the method and the examples of its implementation were presented and discussed in detail. In June 2006, following the meeting in Baltimore, Dr Jose Martines, the representative of the World Health Organization’s Child and Adolescent Health Department (WHO CAH) arranged a meeting in Geneva, Switzerland, where he commissioned a series of 5 CHNRI exercises that would be co–ordinated by WHO CAH and focus on research priorities for five major causes of child deaths: childhood pneumonia, diarrhea, neonatal infections, preterm birth/low birth weight and birth asphyxia. Those exercises were going to be well aligned with UN’s Millennium Development Goal 4 – a political commitment made by world’s nations to reduce global child mortality by two thirds between 1990 and 2015. This was the first major uptake of the CHNRI method by an international organization.

Further refinements of the CHNRI method were introduced based on the feedback received following the presentations at the *International Conference on Priorities in Health Care* in Toronto, Canada, in September 2006 and at the *10^th^ Annual Meeting of the Global Forum for Health Research* in Cairo, Egypt. A parallel session on priority setting in health research investments was organized by CHNRI at the latter conference, with outstanding secretarial support from Ms Carolina Cueva Schaumann, the CHNRI Secretary. The steering committee for the development of the CHNRI methodology approved the publication of the method, allowing for introduction of all the feedback received to date. I led the writing of a series of four papers that described the CHNRI method. I also presented the final revision of the CHNRI method at the *11^th^ Annual Meeting of the Global Forum for Health Research* in Beijing, China, in October 2007. The first two papers of the series that introduced the CHNRI method were published in parallel with the Beijing meeting, in October 2007 [3,4], with the remaining two following in June 2008 [5] and December 2008 [6]. In preparing the four papers, I received large help from Professors Robert E. Black, Shams El Arifeen and Harry Campbell, and further assistance from Drs Lydia Kapiriri, Jennifer Gibson, Mickey Chopra, Kit Yee Chan, Mary Ann Lansang, Ilona Carneiro, Shanthi Ameratunga, Alexander C. Tsai, Mark Tomlinson and Sonja Y. Hess.

An important recognition of the CHNRI method came with an invitation from the World Health Organization’s Cluster on Information, Evidence and Research (IER), its Department for Research Policy and Cooperation (RPC) and the Special Programme for Research and Training in Tropical Diseases (TDR). Those WHO Clusters and Departments convened a workshop in April 2008 to review the available priority setting methodologies for health research. I presented the CHNRI method, which received endorsement for the uptake at the national level through the meeting’s official report. Results of this meeting were later summarized and reported by Tomlinson et al. [7]. I also gave two plenary presentations on the CHNRI method at the *12^th^ Annual Meeting of the Global Forum for Health Research* in Havana, Cuba, in November 2009 and *XIX World Congress of Epidemiology* in Edinburgh in August 2011 [8], where the method was presented to large international audiences and its uptake enhanced.

With a considerable uptake and more than 50 published examples of implementation to date, the CHNRI method is now widely used in many international organisations and professional societies for setting health research priorities. Two recent reviews showed that the CHNRI method has become the dominant approach to setting health research priorities in the global biomedical literature over the past decade [9,10]. Its applications have been helpful in promoting better balance between investments in fundamental research, translation research and implementation research.

## Setting health research priorities: universal challenges and CHNRI’s key conceptual advances

For anyone interested in setting health research priorities at any level, I recommend several comprehensive reviews of the principles, methods, approaches and tools [3,7–11]. Based on those readings, it should become apparent that the CHNRI method proposed its own definition of health research. In CHNRI method’s conceptual framework, “health research” should be regarded as a process that begins with a research question and undertaken to generate new knowledge that will eventually be translated and/or implemented to reduce the existing disease burden (or other health–related problem) in the population [5].

Based on the above definition of health research, the group that developed the CHNRI method identified a considerable number of challenges that will be inherent to any process of setting health research priorities (**Table 1**). In attempts to address those challenges, the CHNRI method introduced three key conceptual advances that led to its increased popularity in comparison to other priority–setting methods and processes.

**Table 1:** A list of twenty “universal challenges” in setting priorities in health research investments, according to the CHNRI method’s conceptual framework [5]

1. Deciding who should be involved in the process of setting health research priorities
2. Defining what constitutes a health research investment option opportunity
3. Defining what constitutes the expected “*return*” on the investment
4. Defining what constitutes a potential “*risk*” of the investment
5. Defining health research, its boundaries, and its levels of “*depth*”
6. Systematic listing of a very large number of competing research investment options
7. Defining what is meant by “*priority setting*” in the context of health research
8. Finding a way to address the uncertainty of health research outcomes
9. Defining criteria relevant to priority setting in health research investments
10. Comparing different instruments of health research using the same criteria
11. Development of a simple quantitative way to rank competing research options
12. Limiting the potential of personal biases to substantially influence the outcome
13. Ensuring that priority–setting process is fully transparent
14. Ensuring that it can be repeated and validated
15. Ensuring that it is flexible and adjustable to all contexts and levels of application
16. Ensuring that it is iterative with a feedback loop, instead of a one–way process
17. Ensuring that it is perceived by the users as legitimate and fair
18. Ensuring that it is simple and intuitive, to become popular among the users
19. Linking quantitative ranks of research options with specific investment decisions
20. Involving stakeholders from the wider community into the process

First, it proposed a solution to the problem of addressing a potentially endless spectrum of research ideas. It proposed a systematic approach to listing a large number of feasible research ideas. To this end, it uses the *“4D framework”* (“description”, “delivery”, “development” and “discovery” research). “*Description*” research includes any proposed health research that would allow researchers to assess the burden of health problems in the population of interest and understand its determinants – ie, negative effects of risk factors and positive effects of delivered health interventions. This is typically achieved through epidemiological research. “*Delivery*” research includes all research questions that allow researchers to optimise health status of the population using the means that are already available. This is typically achieved through implementation research, operations research and/or health policy and systems research. “*Development*” research is focused on improving health interventions that already exist, but could be made more effective, affordable or sustainable. Finally, “*discovery*” research includes all research questions that would lead to innovation, ie, generation of new knowledge to develop entirely new health interventions.

Within each of those four main “*instruments*” of health research – the four D’s – research questions of different “depth” could be posed: very broad “*research avenues*” (which correspond to research fields), more specific “*research options*” (which correspond to a typical research program of about 5 years in duration), and very specific “*research questions*” (which correspond to a title of a typical research paper). Based on this framework, a very large number of proposed research ideas can be systematically assembled and prepared for prioritization (**Table 2**).

**Table 2:** Child Health and Nutrition Research Initiative’s (CHNRI) proposed framework for systematic listing of research ideas in health research, which takes into account the “instruments” of health research (rows) and the “depth” of proposed research ideas (columns)

Research instrument	Research avenue	Research option	Research question
“Description”: research to assess the burden of health problem (disease) and its determinants, ie, negative effects of risk factors and positive effects of delivered health interventions	• Measuring the burden • Understanding risk factors (in terms of their relative risks) • Measuring prevalence of exposure to risk factors • Evaluating the efficacy and effectiveness of interventions in place • Measuring prevalence of coverage of interventions in place	Many research options within each of the avenues; research options should correspond to a research program of up to 5 years in duration	Specific research questions within each of the research avenues should correspond to the title of individual research papers
“Delivery”: research to assist in optimising of the health status of the population using the means that are already available	• Health policy analysis • Health system structure analysis • Financing/costs analysis • Human resources • Provision/infrastructure • Operations research • Responsiveness/recipients		
“Development”: research to improve health interventions that already exist, but could be improved	• Improving existing interventions (their affordability, deliverability, sustainability, acceptability, etc.)		
“Discovery”: research that leads to innovation, ie, entirely new health interventions	• Basic, clinical, and public health research to advance existing knowledge to develop new capacities • Basic, clinical, and public health research to explore entirely novel ideas to develop new capacities		

The second key conceptual advance was defining the context and criteria for prioritization among many research ideas based on a sound framework. The five “*standard*” components of the context in which priority–setting is taking place are the population of interest, the disease burden of interest, geographic limits, time scale and the preferred style of investing with respect to risk (**Table 3**). Depending on who the funders are – government, private sector (eg, pharmaceutical industry and/or biotechnological industry), or philanthropic foundations – their choices of the target population and the health problem of interest, geographic limits, time scale and attitude to risk may be very different. Thus, the elements of the context need to be carefully defined and transparently communicated to scorers before the CHNRI prioritization exercise takes place.

**Table 3:** Elements of the context in which health research prioritization takes place; they need to be clearly defined and communicated to invited technical experts prior to listing and scoring health research ideas

(i) **Population of interest** This element of the context defines the main groups in the society whose health problems are being addressed through health research priority setting.
(ii) **Disease, disability, and death burden** This element of the context defines what is known about the burden of disease, disability, and death that will be addressed by supported health research – e.g., can it be measured and quantified (in disability–adjusted life years–DALYs – or in some other way).
(iii) **Geographic limits** This element of the context defines boundaries in terms of space, which may be global, regional, national, sub–national, etc.
(iv) **Time scale** This element of the context defines the level of urgency, ie, in how many years are the first results of the proposed research expected (they may be defined as reaching the endpoints of the research process, or translating and implementing them, or achieving detectable disease burden reduction).
(v) **Preferred style of investing** This element of the context defines investment strategy in health research with respect to risk preferences; it defines whether most of the funding would support a single (or a few) expensive high–risk research ideas (eg, vaccine development), or will the risk be balanced and diversified between many research options which will have different levels of risk and feasibility.

Once the context was carefully defined according to **Table 3**, and many competing research ideas systematically categorised using the “*4D framework*” in **Table 2**, the next challenge was finding an optimal set of criteria that could distinguish and discriminate between the proposed research ideas, expose their key strengths and weaknesses and assign them an overall “value” according to which they could all be ranked and compared between each other. The chosen set of criteria should be aligned with the guiding principle of the CHNRI method – to expose the potential of many competing health research ideas to reduce disease burden and inequities that exist in the population in a feasible and cost–effective way.

**Table 4** shows a larger number of the possible criteria that could be used to discriminate between the values of any two (or more) competing research ideas. Using such a large number of criteria is clearly impractical, and many of them overlap to a degree and capture similar information about the proposed research idea. Based on CHNRI’s definition of the process of health research, as described earlier in the text, and the likelihood of this process to progress from one stage to another, the five “standard” criteria proposed by the CHNRI method for prioritization between research ideas are: (i) answerability, (ii) effectiveness, (iii) deliverability, (iv) maximum potential for disease burden reduction and (v) the effect on equity.

**Table 4:** Some of the possible priority–setting criteria (and related questions) proposed by Child Health and Nutrition Research Initiative (CHNRI) that can be used to discriminate between any two (or more) health research ideas to set research priorities; the outcomes of the application of different criteria will necessarily conflict each other

**Answerability?** (some health research ideas will be more likely to be answerable than the others)
**Attractiveness?** (some health research ideas will be more likely to lead to publications in high–impact journals)
**Novelty?** (some health research ideas will be more likely to generate truly novel and non–existing knowledge)
**Potential for translation?** (some health research ideas will be more likely to generate knowledge that will be translated into health intervention)
**Effectiveness?** (some health research ideas will be more likely to generate/improve truly effective health interventions)
**Affordability?** (the translation or implementation of knowledge generated through some health research ideas will not be affordable within the context)
**Deliverability?** (some health research ideas will lead to / impact health interventions that will not be deliverable within the context)
**Sustainability?** (some health research ideas will lead to / impact health interventions that will not be sustainable within the context)
**Public opinion?** (some health research ideas will seem more justified and acceptable to general public than the others)
**Ethical aspects?** (some health research ideas will be more likely to raise ethical concerns than the others)
**Maximum potential impact on the burden?** (some health research ideas will have a theoretical potential to reduce much larger portions of the existing disease burden than the others)
**Equity?** (some health research ideas will lead to health interventions that will only be accessible to the privileged in the society/context, thus increasing inequity)
**Community involvement?** (some health research ideas will have more additional positive side–effects through community involvement)
**Feasibility?** (some health research ideas will be unlikely to lead to translation at the current stage of knowledge)
**Relevance?** (some health research ideas will be more relevant to the context than the others)
**Fills key gap?** (some health research ideas will be more likely to fill the key gap in knowledge that is required for translation and/or implementation than the others)
**Cost?** (some research ideas will require more funding than the others)
**Fundability?** (some research ideas will be more likely to receive funding support within the defined context than the others)
**Alignment with political priorities?** (some research ideas will be more likely to be aligned with contemporary political priorities than the others)
**Likelihood of generating patents/lucrative products?** (some research ideas will have greater likelihood of generating patents or other potentially lucrative products, thus promising greater financial return on investments, regardless of their impact on disease burden)

However, an advantage of the CHNRI method is that both the elements of the context and the number and the composition of the criteria can be flexibly changed to meet the specific needs of each priority–setting exercise. Further elements may be added to the context description, or some of the proposed ones can be dropped or replaced. The same is true for the priority–setting criteria, and I encourage the users of the method to take advantage of this flexibility to meet the goals of their specific exercise. I believe that the CHNRI method owes its uptake and implementation in a large part to its flexibility, as it can be readily tailored to many different contexts and purposes.

For example, in different contexts addressing of the “*answerability*” criterion may also require a separate assessment related to the ethics of the proposed research idea, an evaluation of the existing research capacity, or an assessment of the likely public acceptance of research results. The “*relevance*” criterion may need to be further refined into criteria that would separately assess effectiveness, deliverability, affordability, sustainability, and whether a critical gap in knowledge is being addressed. The “*maximum potential impact on the burden*” will occasionally not only assess the quantity of potential burden reduction, but also its quality – ie, whether this reduction is targeting those most heavily affected or underprivileged in the population [5]. **Table 4** lists a number of possible criteria that can be used for setting priorities between different research investment options and questions about each option that could address these criteria well.

The third key conceptual advance of the CHNRI method relates to the problem of consensus development and agreement on the priorities among many proposed research ideas. Before the introduction of the CHNRI method, a typical consensus development process would involve the so–called Delphi method [12,13]. This process would typically require background reading, followed by the first round of discussions among relatively small groups of experts. Expert interactions and the opportunities for the experts to influence one another defined the process of consensus development. There would usually be a step where a feedback would be provided to experts, which would further influence their independent opinion, followed by the second round of discussions. Eventually, the groups would reach a consensus on research priorities. The problem with this process was that it could not be considered transparent, replicable or democratic, because at each stage there was a large opportunity for the managers of the process, or individual participants with strong opinions, to influence all other participants.

At the time of the development of the CHNRI method, the rise of information technologies and online communication enabled the new approach to developing consensus among a larger group of people through so–called “crowdsourcing”. It was proposed that simply reaching out to a large number of people and assessing their collective opinion (in this case, optimism toward a large number of research ideas to fulfil the specific priority–setting criteria) may result in surprisingly accurate predictions that would typically surpass any individual’s expert judgement [14]. However, there were several requirements that needed to be met to ensure that the collective opinion would indeed be useful. Those included diversity of opinion (meaning that each participant should have his/her private information), independence of participants (meaning that participants’ input wouldn’t be influenced by the opinion of other participants), decentralization (meaning that participants would be diverse and able to draw on any local knowledge) and aggregation (meaning that a mechanism would be available for collecting many individual opinions and turning them into a collective opinion). The rise of information technologies–based communication allowed to collect information from a large number of international experts in global health quickly and efficiently, with all the above requirements met.

Thanks to this advance, the CHNRI method proposed a radically different approach to consensus development from the Delphi process. In both methods input from experts is required, and the invited experts have the same background characteristics. However, the CHNRI method collects opinion from many international experts through their e–mail input, no background reading is required, and no discussions or interactions would occur between many participating experts. Feedback on their collective opinion could still be returned to participants, but there would not be a need for a step where consensus would need to be developed, because a simple quantitative analysis of the received input would turn the information obtained from each expert into a “collective” result, which would belong to every participant, but no single participant would have a chance of influencing any substantial portion of it. Then, the areas of greater or smaller consensus could be identified through agreement statistics analysis of the input, without a need for a second round of discussion.

Thus, the CHNRI method innovated the process of consensus development through measuring collective optimism of a larger number of international experts on each research idea and each criterion. This was done through consulting a larger group of experts and using a very simple scoring system, where they only needed to say whether they thought that the research idea was likely, or not, to meet the priority–setting criterion within the specified context. This enabled the use of the knowledge of many experts in the field, “visualising” their collective opinion and presenting the list of many research ideas with their ranks, based on an intuitive score that ranges between 0% (absolutely no optimism) and 100% (where everyone is optimistic). In this way, the knowledge of a larger number of international experts is used, through “crowdsourcing”, to discriminate between competing research options based on strictly defined criteria and the collective optimism toward compliance of each research option with each criterion. Such approach limits the potential of individual personal biases to substantially influence the outcome, which was identified as a major challenge that needed to be addressed.

The proposed conceptual advance in the CHNRI method ensured that the scoring experts provided their input independently of each other, and that the final scores for each competing research option were obtained and computed in a highly structured, transparent, systematic and replicable way. Through application of agreement statistics methods, the CHNRI method could then also identify and expose controversial issues (ie, responses with a large variation in scores among experts). Finally, the proposed method promised to generate a large amount of useful information for funders of research and research communities alike, by “visualising” the collective opinion of many leading experts on many research ideas and their key components.

The above characteristics of the CHNRI method also dealt with several other universal challenges. The flexibility in the choice of context and criteria ensured that the method would be adjustable to all contexts and areas of application [5]. It also envisioned a “feedback loop”, because the process of priority setting could be repeated after certain periods of time, allowing the priorities to change with the changing context. Transparency and clarity of the proposed steps of the CHNRI method were intended to ensure that it is perceived as legitimate and fair by its users [15,16].

## CONCLUSIONS

The CHNRI method measures collective optimism of a larger number of researchers toward various components of many proposed research ideas, within an agreed context and using the agreed criteria. Because of this process, a large number of health research ideas would receive their intermediate and overall “priority scores”, which will be in a quantitative form, ranging from 0 to100%. This should provide a large amount of useful information to many funders, researchers and stakeholders alike. Advantages and disadvantages of each research idea should become transparent through this process, which would be based on a “democratic” assessment [5].

A common misconception in the early days of the CHNRI method development and implementation was that the CHNRI process would be telling the funders where to invest their resources. However, this is not what the CHNRI method does to any extent. It is designed to merely present a very large amount of information to the funders on many research ideas, including their strengths and weaknesses. In a way, this is not much different from the information available on the performance of various companies that are potential investment options in the stockmarket. The CHNRI process should simply allow the funders of health research to choose from many research ideas based on a lot of information that the expert group provided on each idea. This should protect funders from risky investments and allow them to develop their own investment style and portfolio [5].

There are further practical advantages of the CHNRI exercise, such as the ease of conducting the exercise over the internet, low cost of planning and conducting the exercise, ease of obtaining information from many experts online, and excellent prospects of publishing the results of each exercise. Modifications of the CHNRI method should also allow prioritization among investments in health care, emerging health technologies, and development assistance for health. The CHNRI method should be of possible use to research funding bodies, international organizations and for–profit companies in setting their own strategic priorities among many different ideas, based on collective knowledge of their most qualified employees or external experts [5].

The CHNRI method is not free from shortcomings and possible concerns over the validity of the process. I will address the most important among those concerns in the following papers of this series through a set of carefully designed experiments into quantitative properties of human collective knowledge and opinion. Those studies should bring more certainty over the components of the CHNRI process that are critical for its validity. This will be followed by definite guidelines for implementation, based on a review of more than 50 exercised conducted to date and their impact on research policy.
